# Imaging findings for 2-[^18^F]fluoro-2-deoxy-D-glucose positron emission tomography/computed tomography in blastic plasmacytoid dendritic cell neoplasm: case series and literature review

**DOI:** 10.3389/fmed.2025.1581760

**Published:** 2025-09-19

**Authors:** Yujing Zhou, Kaiyue Li, Xin Jin, Lujie Yuan, Hang Zhou, Xin Li

**Affiliations:** ^1^Department of Nuclear Medicine, Qilu Hospital of Shandong University, Jinan, Shandong, China; ^2^Imaging Center, Jinan Third People's Hospital, Jinan, Shandong, China

**Keywords:** blastic plasmacytoid dendritic cell neoplasm, ^18^F-FDG PET/CT, hematologic malignancy, imaging manifestations, prognosis

## Abstract

Blastic plasmacytoid dendritic cell neoplasm (BPDCN) is a rare hematologic malignancy with a poor prognosis. The value of 2-[^18^F]fluoro-2-deoxy-D-glucose positron emission tomography/computed tomography (^18^F-FDG PET/CT) in BPDCN has not been clarified. This study aimed to investigate the imaging findings of ^18^F-FDG PET/CT in patients with BPDCN based on cases from our institution and the literature. The clinical and radiological data were obtained from five patients with BPDCN from our institution between March 2014 and July 2023. Additionally, we complemented our dataset with 13 cases derived from the studies published in English to ascertain the potential efficacy of ^18^F-FDG PET/CT scan in identifying this malignancy. Information collected included age, sex, extent of lesion involvement, biopsy site, karyotype, immunophenotype, treatment, prognosis, and ^18^F-FDG PET/CT-features. A total of 18 cases of BPDCN featuring PET/CT manifestations were assessed. We observed considerably increased ^18^F-FDG uptake in lesions of all 18 cases [maximum standardized uptake value (SUV_max_), 9.1; range, 1.5–9.1]. The positive findings of ^18^F-FDG PET/CT mainly included skin (11/18), lymph nodes (9/18), bone (4/18), and spleen (2/18). Except for these organs, abnormal ^18^F-FDG uptake lesions were detected in the lung and breast. The roles of ^18^F-FDG PET/CT in our study were initial staging (18/18), selection of biopsy site (5/18), and treatment evaluation (7/18). Prognostic data were available in 16 patients. The median overall survival (OS) in this cohort was 12.0 months, and the median follow-up time was 10.0 months. Among these, 10 cases reported SUV_max_ of lesions at the same time. Five out of eight patients with SUV_max_ > 2.5 died within 2 months of diagnosis, whereas two other cases with SUV_max_ < 2.5 survived within 10 and 34 months of follow-up. The data from our case series and those from the literature demonstrated the potential utility of ^18^F-FDG PET/CT in diagnosis, staging, prognosis, and treatment follow-up of BPDCN. Early identification of this rare malignancy on imaging can expedite diagnosis and facilitate early treatment.

## Introduction

Blastic plasmacytoid dendritic cell neoplasm (BPDCN) is a rare hematologic malignancy with a poor prognosis (1). BPDCN was classified as a distinct clinical entity within the myeloid class of neoplasms in 2016 by the World Health Organization (2). The disease may occur at any age, but most patients are in their sixth or seventh decade of life (3). BPDCN commonly presents as cutaneous lesions, systemic dissemination involving lymph nodes, bone marrow, and extranodal lesions. Laribi et al. (4) reported 398 patients diagnosed with BPDCN. The involvement of the skin, bone marrow, lymph nodes, and peripheral blood at diagnosis was observed in 89, 62, 39, and 15% of cases, respectively. Although infiltrations of extranodal localizations were rare, cases involving the central nervous system (5), ocular adnexa (6), lung (7), breast (8), and uterus (9) were reported. Pathological diagnosis of BPDCN relies on the expression of molecular markers CD4, CD56, and CD123, along with negativity for lineage-specific markers. Furthermore, other markers restricted to plasmacytoid dendritic cells can be positive. However, certain rare cases may lack CD56 (4). Given that the treatment options chosen vary depending on the location of the lesion, it is vital to evaluate the overall condition of patients and the extent of disease involvement at first diagnosis.

The development of positron emission tomography (PET) was a milestone in the development of modern imaging technology. At present, it, combined with computed tomography (CT), reveals metabolic function through the uptake of the radionuclide-labeled probe (10). Increasing evidence indicates that 2-[^18^F]fluoro-2-deoxy-D-glucose PET/CT (^18^F-FDG PET/CT) is feasible for assessing pretreatment staging, detecting organ infiltration, and evaluating the treatment response in hematologic malignancy (11). As BPDCN involves multiple systems, ^18^F-FDG PET/CT may offer an ideal method to observe the glucose metabolism of the whole body in a single examination. Although ^18^F-FDG PET/CT is used in the clinical treatment of BPDCN, few scholars have reported PET/CT features of the disease.

This study presented five cases of BPDCN from our institution and discussed the potential utility of ^18^F-FDG PET/CT in BPDCN in a literature review. It is pertinent to underscore that this retrospective study was carried out in accordance with the Declaration of Helsinki (2000) of the World Medical Association. The study received approval from the ethics committee of Qilu Hospital at Shandong University. Informed consent forms were signed by all participants or their next of kin. We aimed to summarize the role of ^18^F-FDG PET/CT in observing organ involvement, guiding biopsies, and assessing the response to chemotherapy in BPDCN treatment.

## Case presentation

### Case report 1

An 82-year-old man visited our dermatological department with complaints of nodular violaceous rash scattered on the skin of his arm, chest, and back for 2 months. His past medical history included hypertension and type 2 diabetes. The physical examination revealed multiple 3.0- to 5.0-cm nodules in his arms, chest, and back. No palpable lymphadenopathy or hepatosplenomegaly was reported. A complete blood count revealed mild normochromic normocytic anemia. No abnormalities were detected in the bone marrow aspirate and trephine biopsy. Skin biopsy from the back confirmed the disease as BPDCN. ^18^F-FDG PET/CT revealed multiple mild FDG-avid cutaneous lesions on the arm, chest, and back with an SUV_max_ of 1.5 (the SUV of the liver was measured as 2.6). The patient is currently receiving the first cycle of chemotherapy with azacitidine and venetoclax.

### Case report 2

A previously healthy 26-year-old woman presented to our dermatological department with a 6-month history of an asymptomatic dark, violaceous, infiltrated nodular lesion on the left cheek. Physical examination revealed no other obvious abnormalities. Hematology and bone marrow examinations were normal. A skin biopsy of the lesion confirmed the disease as BPDCN. ^18^F-FDG PET/CT revealed a 3.0 × 5.5 cm lesion on the left cheek with moderate FDG accumulation at an SUV_max_ of 2.8 (the SUV of the liver was measured as 3.0). Although the patient received multiple chemotherapeutic regimens, she experienced bone marrow infiltration and died 1 year after diagnosis.

### Case report 3

A 51-year-old woman presented with left cervical lymphadenopathy for more than 2 months, which was diagnosed as BPDCN on biopsy. She had undergone a hysterectomy for fibroids 10 years ago. Physical examination revealed multiple enlarged lymph nodes on the bilateral sides of the neck and clavicle areas. A complete blood count was normal. No neoplastic cells were detected in the bone marrow. ^18^F-FDG PET/CT revealed moderate-to-high FDG-avid cervical and clavicular lymphadenopathy with an SUV_max_ of 6.8 (SUV of the liver was measured as 3.7). She experienced post-chemotherapeutic bone marrow suppression after one cycle of vincristine, daunorubicin, cyclophosphamide, L-asparaginase, and prednisone (VDCLP) treatment and died 10 days after starting therapy.

### Case report 4

**Figure 1:**
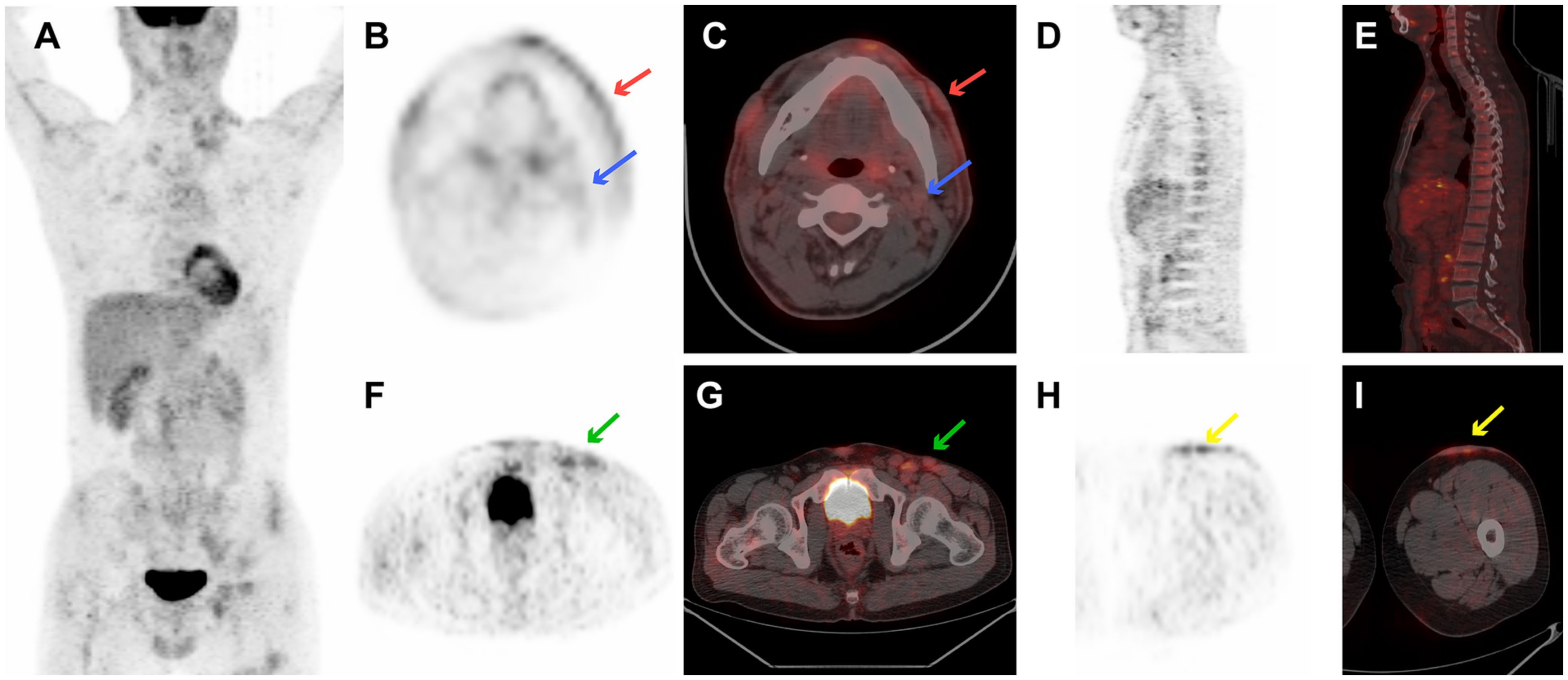
^18^F-FDG PET/CT images of case 4 revealed multiple mild-to-moderate FDG-avid cutaneous lesions on the head, neck, chest, back, and left femoral region with an SUV_max_ of 2.6. There are supra- and infra-diaphragmatic abnormally enlarged lymph nodes with an SUV_max_ of 3.7. Mild FDG-avid of trunk bone was detected with a SUVmax 1.5. **(A)** PET maximum intensity projection (MIP), **(B)** transverse PET image of cutaneous lesion of cheek (red arrows) and cervical lymph nodes (blue arrow), **(C)** transverse fused PET/CT image of cervical cutaneous lesion of cheek (red arrows) and cervical lymph nodes (blue arrow), **(D)** sagittal PET image of bone, **(E)** sagittal fused PET/CT image of bone, **(F)** transverse PET image of left inguinal lymph nodes (green arrow), **(G)** transverse fused PET/CT image of left inguinal lymph nodes (green arrow), **(H)** transverse PET image of cutaneous lesion in left femoral region (yellow arrows), and **(I)** transverse fused PET/CT image of cutaneous lesion in left femoral region (yellow arrows).

A 36-year-old man presented with a 5-month history of a nodular violaceous rash on the skin and multiple lymphadenopathies. He had no specific medical history. Physical examination revealed multiple nodules over his head, neck, chest, and back, and in the left femoral region, together with multiple enlarged lymph nodes in the bilateral neck, bilateral armpits, left groin, and bilateral supraclavicular areas. The complete blood count revealed pancytopenia (hemoglobin = 58 g/L, white blood cell = 3.2 × 10 ^9^/L, and platelet = 4 × 10 ^9^/L).^18^F-FDG PET/CT image is illustrated in Figure 1. Figure 1 shows the ^18^F-FDG PET/CT images of case 4, with disseminated cutaneous, lymph nodes, and extranodal localization disease (Figure 1A). ^18^F-FDG PET/CT revealed multiple mild-to-moderate FDG-avid cutaneous lesions on his head, neck, chest, and back, and in the left femoral region with an SUV_max_ of 2.6 (the SUV of the liver was measured at 2.7). Supra- and infra-diaphragmatic abnormally enlarged lymph nodes were seen in cervical, supraclavicular, axillary, mediastinal, retroperitoneal, and left bilateral inguinal areas with an SUV_max_ of 3.7 (Figures 1B,C,F–I). Mild FDG-avid in the trunk bone was detected with an SUV_max_ of 1.5 (Figures 1D,E). This patient underwent a skin biopsy, cervical lymph node biopsy, and bone marrow examination, all of which indicated BPDCN. He died from gastrointestinal bleeding due to abnormal coagulation function 15 days after diagnosis.

### Case report 5

**Figure 2:**
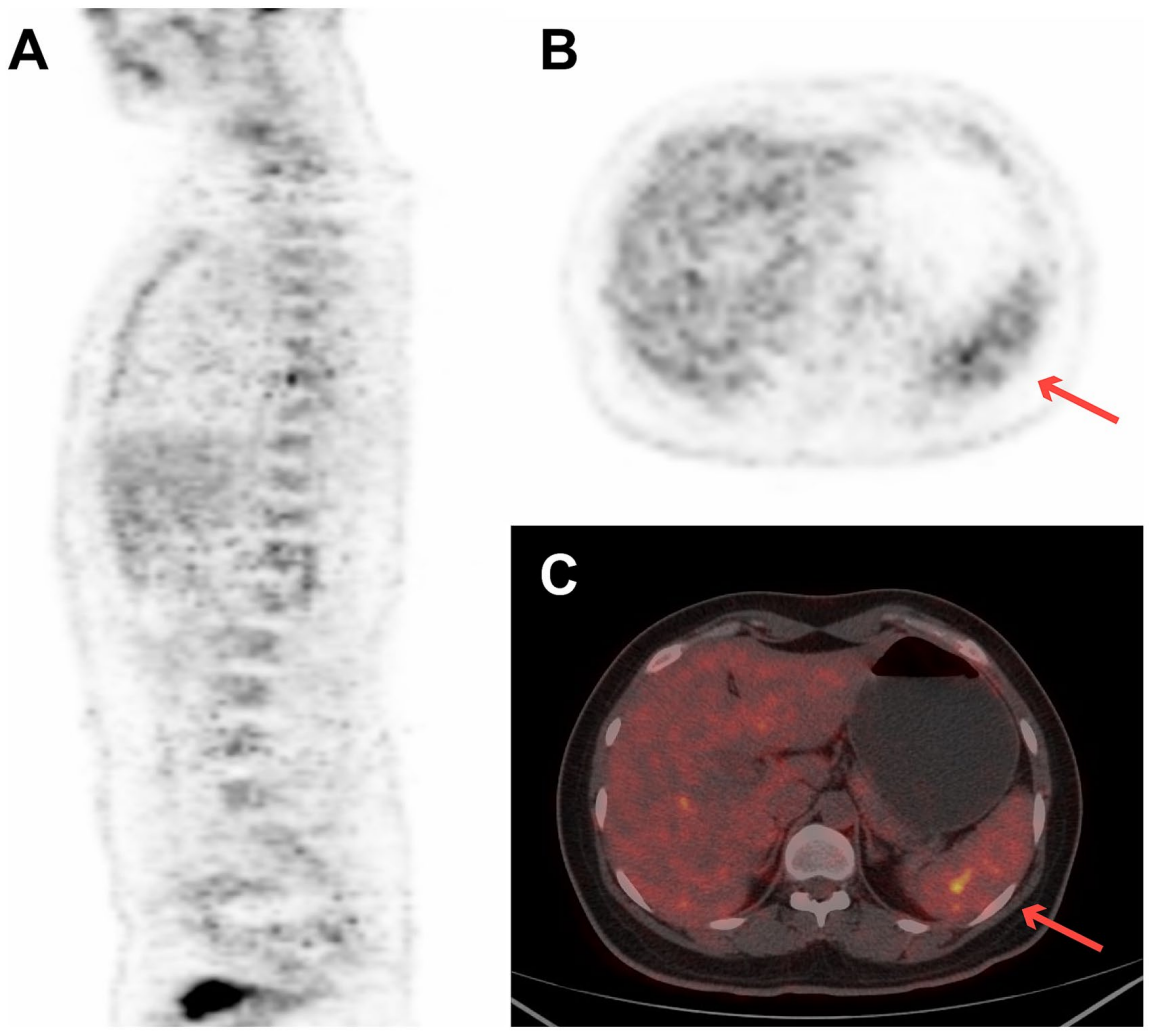
^18^F-FDG PET/CT images of case 5 revealed high FDG-avid of the spleen (SUVmax 4.4) and bones (SUVmax 9.1). **(A)** Sagittal PET image of bone, **(B)** transverse PET image of spleen (red arrow), and **(C)** transverse fused PET/CT image of spleen (red arrow).

A 27-year-old woman with a 2-week history of back pain was admitted to our orthopedics department. The complete blood count revealed thrombocytopenia (platelet count = 58 × 10^9^/L). Physical examination revealed scattered ecchymosis on the skin. Figure 2 shows images of case 5 with bone marrow and spleen involvement. ^18^F-FDG PET/CT revealed high FDG-avid lesions of the bones (SUV_max_, 9.1, Figure 2A) and spleen (SUV_max_, 4.4, Figures 2B,C). No clear lesions were detected at any other sites. This patient underwent a bone marrow examination, and the disease was confirmed as BPDCN. She died of disseminated intravascular coagulation 20 days after diagnosis.

## Discussion

In this article, we present five cases of BPDCN diagnosed with ^18^F-FDG PET/CT assistance. Subsequently, we performed a systematic literature review using the Web of Science and PubMed databases between January 2000 and July 2023. Combination search terms included “blastic plasmacytoid dendritic cell neoplasm,” “^18^F-fluorodeoxyglucose-positron emission tomography/computed tomography,” “PET/CT,” and “PET.” The inclusion criteria were: (a) patients diagnosed with BPDCN confirmed by pathologic examination of skin and lymph node or bone marrow biopsy and (b) patients underwent ^18^F-FDG PET/CT for initial staging at diagnosis. The exclusion criteria were patients with a previously diagnosed malignant condition. Finally, 12 articles (including 13 cases) were retrieved (7, 8, 12–21) from the databases.

From March 2014 to July 2023, five patients (cases 1–5) were diagnosed with BPDCN and underwent ^18^F-FDG PET/CT for initial staging in Qilu Hospital of Shandong University. We identified 12 studies (including 13 cases) describing ^18^F-FDG PET/CT features at diagnosis of BPDCN (7, 8, 12–21). Data collected from our cases and cases in the literature included age, sex, extent of lesion involvement, biopsy site, immunophenotype, treatment, prognosis, and ^18^F-FDG PET/CT features. Treatments were categorized as acute myeloid leukemia (AML)-like, acute lymphoblastic leukemia (ALL)-like, or non-Hodgkin lymphoma (NHL)-like chemotherapy regimens, peripheral blood stem cell transplant (PBSCT), radiotherapy, and new drug (SL401) or palliative treatment. Prognosis was defined as overall survival (OS). OS was defined as the time from the first diagnosis of BPDCN to the last follow-up or death. SPSS 24.0 software (SPSS, Inc., Chicago, IL) was used for statistical analyses. The mean age of the cohort is expressed as the median (25–75th percentile). The survival curves were performed using the Kaplan–Meier method.

BPDCN, an extremely rare and aggressive tumor, is derived from precursor plasmacytoid cells. Most patients present with cutaneous lesions, followed by systemic dissemination involving lymph nodes and extranodal localizations (4). Therefore, it is necessary to seek accurate imaging methods for detecting systemic involvement. Given that a single examination can observe lesions and their metabolism throughout the whole body, ^18^F-FDG PET/CT has been considered a reliable method for detecting hematopoietic malignancy and plays a vital role in staging and clinical decision-making, especially in the treatment of lymphoma (22). For BPDCN, ^18^F-FDG PET/CT is an effective examination method because of the heterogeneity of the affected organs.

Together with our 5 cases, 18 cases of BPDCN containing PET/CT manifestations were reported (7, 8, 12–21). The clinical features are listed in Table 1. The study included 11 male and 7 female patients. The mean age of our cohort was 35.5 years (17.5–74.8), ranging from 8 to 87 years. The disease was limited to skin (3/18), cutaneous isolated and lymph nodes (1/18), cutaneous isolated and non-cutaneous extranodal localization (1/18), disseminated with cutaneous localization (3/18), disseminated with lymph nodes (1/18), disseminated with lymph node and non-cutaneous extranodal localization (2/18), disseminated with cutaneous, lymph node, and extranodal localization (5/18), and disseminated with non-cutaneous extranodal localization (2/18). Karyotype data were available in five patients, and only one presented with a calreticulin (CALR, Exon9) mutation. The CALR-mutated case (case 14) showed particularly aggressive PET/CT features (multifocal lesions, SUVmax 9.1). This aligns with emerging evidence of molecular heterogeneity in BPDCN (23), though our dataset is underpowered for formal correlation analysis. Furthermore, 15 of 18 patients provided phenotypic information, and 8 of them had a combination of CD4+, CD56+, and CD123+. Three of them revealed a negative expression of CD56.

**Table 1:** Clinical characteristics of 18 patients with BPDCN.

Patient no.	Age/sex	Forms	Biopsy site	Karyotype	Immunophenotype					Type of treatment	Prognosis	Ref.
					BPDC markers	T-lymphoid and natural killer cell markers	B-lymphoid markers	Myeloid and monocyte markers	Immature and other markers			
1	82/M	Disseminated with cutaneous localization	Skin (back)	NA	CD123(+), CD56(+), CD4(+)	CD2(−), CD3(−), CD8(−)	CD20(−)	MPO(−), CD117(−)	CD30(−), TdT(−)	AML-like	PFS: 10 m.	Case 1
2	26/F	Cutaneous isolated	Skin (left cheek)	NA	CD123(+), CD56(+), CD4(+)	CD2(+)	CD79a(−) CD20(−)	NA	CD30(−), CD34(−), CD43(+), TdT(−)	NHL-like	OS:1.2 m.	Case 2
3	51/F	Disseminated with lymph nodes	Left cervical lymphadenopathy	NA	CD123(−), CD56(+), CD4(+)	CD3(−), CD5(−)	CD20(−), CD79a(−)	NA	CD10(−), Bcl-2(+), TdT(+)	ALL-like	OS: 1.6 m.	Case 3
4	36/M	Disseminated cutaneous, lymph nodes, and extranodal localization	skin (chest), bone marrow, left inguinal lymph node	NA	CD123(+), CD56(−), CD4(+)	CD3(−), CD5(−)	CD79α(+)CD20(−)	MPO(−)	CD43(+), S100(−), TdT(−)	Palliative	OS: 0.5 m.	Case 4
5	27/F	Disseminated non-cutaneous extranodal localization	Bone marrow	NA	NA	NA	NA	NA	NA	Palliative	OS: 1 m.	Case 5
6	34/M	Disseminated with cutaneous localization	Cutaneous mass (left lower leg)	NA	CD123(+), CD56(+), CD4(+)	CD3(−)	CD20(−)	MPO(−)	CD30(−)	NA	NA	(12)
7	9/M	Disseminated cutaneous, lymph nodes, and extranodal localization	cutaneous mass (right foot)	NA	CD56(+), CD4(+)	NA	NA	NA	NA	NA	CR after 3.8 months of follow-up	(13)
8	35/F	Disseminated lymph nodes and non-cutaneous extranodal localization	Axillary lymph node	NA	CD56(−), CD4(+)	CD3(−)	CD20(−), CD79a(−), CD138(−)	MPO(−)	CD30(−), CD43(+), TdT(−)	Palliative	OS: 0.3 m.	(14)
9	19/M	Cutaneous isolated	Left elbow	NA	CD56(+), CD4(+)	CD3(−)	CD20(−)CD79a(−) CD138(−)	CD68(+), MPO(−)	CD30(−), CD34(−), CD43(+), TdT(+)	ALL-like	CR after 9 months of follow-up	(14)
10	47/F	Cutaneous isolated and non-cutaneous extranodal localization	Right cheek, breast	Normal	CD56(+), CD4(+)	CD3(−)	CD20(−)	CD68(+), MPO(−)	CD10(−), CD34(−), Bcl2(+), TdT(+)	ALL-like+ PBSCT	CR after 34 months of follow-up	(8)
11	87/F	Disseminated lymph nodes and non-cutaneous extranodal localization	Bone marrow	Normal	CD123(+), CD56(−), CD4(+)	cCD3(−), CD2(+), CD7(+)	NA	CD36(+)	CD34(−) CD38(−) HLA-DR (+)	AML-like	OS: 6 m.	(15)
12	82/F	Disseminated non-cutaneous extranodal localization	Lung, bone marrow	Normal	CD123(+), CD56(+), CD4(+)	CD3(−)	NA	NA	CD45RA(+), D34(−), CD38(+), CD43(+)	Multiple chemotherapeutic regimens	OS: 3 m.	(7)
13	13/M	Cutaneous isolated	Left cheek	NA	CD123(+), CD56(+), CD4(+)	CD3(−), CD8(−)	CD20(−)PAX5(−)	MPO(−)	CD10(−), TdT(−)	ALL-like	Good response after 9 months of follow-up	(16)
14	67/M	Disseminated cutaneous, lymph nodes, and extranodal localization	Skin(chest), inguinal lymph node, bone marrow	CALR (Exon9) L367fs*46	CD123(+), CD56(+), CD4(+)	Granzyme B(−), CD2(−), CD7(−)	CD20(−)CD138(−)	MPO(−), CD68(−), CD117(−)	CD3(−), CD34(−), CD43(+), TdT(+)	ALL-like	OS:12 m.	(17)
15	9/M	Cutaneous isolated and lymph nodes	Skin (left forearm)	NA	CD123(+), CD56(+), CD4(+)	CD1a(−), CD3(−), CD5(−), CD7(−), CD8(−)	CD79a(+)PAX5(−), CD20(−),	MPO(−), CD14(−), CD15(−), CD33(−), CD68(−), CD117(−)	CD10(−), CD34(−), CD45(+), TdT(+)	NHL-like+ALL-like	CR after 15 months of follow-up	(18)
16	8/M	Disseminated cutaneous, lymph nodes, and extranodal localization	Skin (left calf)	NA	NA	NA	NA	NA	NA	chemotherapy	Good response after 4 months of follow-up	(19)
17	77/M	Disseminated with cutaneous localization	Skin (left forearm and right shoulder)	Normal	CD123(+), CD56(+), CD4(+)	CD1a(−)	CD20(−)	MPO(−)	S-100(−)	Radiotherapy	NA	(20)
18	74/M	Disseminated cutaneous, lymph nodes, and extranodal localization	NA	NA	NA	NA	NA	NA	NA	SL401	CR after 3 months of follow-up	(21)

NA, not available; RFS, relapse-free survival; OS, overall survival; AML, acute myeloid leukemia; ALL, acute lymphoblastic leukemia; NHL, non-Hodgkin lymphoma; PBSCT, peripheral blood stem cell transplant; CR, complete remission.

Although the clinical features and pathology establish the diagnosis of BPDCN, it is crucial to recognize the PET/CT features that can characterize the extent of disease involvement. However, no study has summarized the imaging manifestations of BPDCN, and evidence of the application value of BPDCN is lacking. A reported international survey of 398 adult patients indicated that 89% of them had skin involvement, either a single mass or multiple scattered skin lesions (4). ^18^F-FDG PET/CT results in BPDCN, including our case series and cases in the literature (7, 8, 12–21), are depicted in Table 2. ^18^F-FDG uptake was mildly to significantly increased in lesions in all 18 cases (SUV_max_, 9.1; range, 1.5–9.1). The positive findings of ^18^F-FDG PET/CT mainly included skin (11/18), lymph nodes (9/18), bone (4/18), and spleen (2/18). Four cases (57.1%) had high uptake of FDG in bone among seven cases with bone marrow infiltration confirmed by bone marrow biopsy. Except for these organs, abnormal ^18^F-FDG uptake lesions were detected in the lung (1/18; SUV_max_, 5.6) and breast (1/18; SUV_max_, 1.9). A summary of SUV_max_ values in ^18^F-FDG PET/CT of BPDCN is depicted in Table 3. For cutaneous isolated lesions, the FDG uptake was moderate-to-high (range, 2.8–6.7). However, for multiple cutaneous lesions, the FDG uptake was mild-to-moderate (range, 1.5–2.6). Moderate-to-high uptake was observed in lymph nodes (range, 2.3–6.8). For two patients with bone marrow infiltration having PET/CT data, the SUV_max_ of bone was 1.5 and 9.1, respectively. In our study, the single mass or skin lesion manifested as high FDG uptake with a range of 2.8–6.7. The FDG uptake level in multiple skin lesions was relatively low, manifested as a low-to-moderate FDG-avid (range, 1.5–2.6). Besides metabolism-related information, the simultaneous CT images also provided a more accurate measurement of lesion depth and thickness than physical examination (24). Moreover, PET/CT could detect deeper lesions, which were otherwise invisible. The combination of the two provided more useful imaging information. However, the resolution of the PET scan may miss patches and thin plaques, and the partial volume effect may lead to the underestimation of the FDG uptake (24). The imaging feature is also non-specific (12); hence, it is sometimes necessary to consider other clinical manifestations comprehensively.

**Table 2:** ^18^F-FDG PET/CT features of BPDCN and role in clinical practice.

Patient no.	^18^F-FDG PET/CT findings at initial (SUVmax)	^18^F-FDG PET/CT for treatment response or recurrence	Role of ^18^F-FDG PET/CT	Ref.
1	Mild FDG-avid cutaneous lesion on the arm, chest, and back (SUVmax 1.5)		Initial staging	Case 1
2	Moderate FDG-avid lesion in the left cheek (SUVmax 2.8)	Resolution of the hypermetabolic lesion in the left cheek (6 weeks after the pretreatment)	Initial staging; Treatment evaluation	Case 2
3	High FDG-avid cervical and clavicular lymphadenopathy (SUVmax 6.8)		Initial staging	Case 3
4	Multiple mild-to-moderate FDG-avid cutaneous lesions on his head, neck, chest, back, and left femoral region (SUVmax 2.6) Moderate-to-high FDG-avid supra- and infra-diaphragmatic abnormally enlarged lymph nodes (SUVmax 3.7) Mild FDG-avid of trunk bone (SUVmax 1.5)		Initial staging Guide biopsies	Case 4
5	Diffuse accumulation in spleen (SUVmax 4.4) High FDG-avid of bone (SUVmax 9.1)		Initial staging Guide biopsies	Case 5
6	Multiple mild FDG-avid cutaneous lesions on the back, with involvement of the left inguinal lymph node Markedly increased FDG-avid subcutaneous mass in the left lower leg (SUVmax 6.7)		Initial staging	(12)
7	FDG avid soft tissue mass on the dorsal aspect of the right foot (SUVmax 4.1) Metastatic disease of the ipsilateral inguinal and iliac lymph nodes Osseous lesion in the contralateral distal femur	Complete interval resolution of the hypermetabolic inguinal and iliac lymphadenopathy and the osseous lesion	Initial staging, Treatment evaluation	(13)
8	Nasal mass, cervical, abdominal, axillary, and mediastinal lymphadenopathy		Initial staging	(14)
9	Metabolically active disease involving the anteromedial aspect of the left elbow		Initial staging	(14)
10	Mild FDG-avid lesion on the right cheek (SUVmax 2.2) Hypermetabolic mass lesion in the right breast (SUVmax 1.9)		Initial staging; Guide biopsies	(8)
11	Supra- and infra-diaphragmatic abnormally enlarged lymph nodes Diffuse accumulation in bone and spleen	Complete metabolic remission (after the third cycle of association)	Initial staging; Treatment evaluation	(15)
12	Elevated FDG uptake in bilateral lungs consistent with interstitial lesions (SUVmax 5.6)		Initial staging Guide biopsies	(7)
13	Hypermetabolic activity localized on the left cheek (SUVmax 4.68)		Initial staging	(16)
14	Multiple mild FDG-avid cutaneous lesions and involvement of lymph nodes and trunk bone		Initial staging; Guide biopsies	(17)
15	Hypermetabolic thickening of the soft tissue in the left forearm and bilateral group 2 cervical lymph nodes Significant metabolic reaction of the left axillary lymph node.	Nearly completely eradicated (following six courses of intensive chemotherapy) No tumor recurrence was detected on PET/CT (15 months after diagnosis).	Initial staging; Treatment evaluation	(18)
16	FDG uptake within multiple enlarged lymph nodes in the left inguinal, left internal iliac, and left para-aortic regions	Resolution of the hypermetabolic lymphadenopathy (4 weeks after the pretreatment).	Initial staging; Treatment evaluation	(19)
17	Accumulation was present in the skin of the left forearm (SUVmax 3.5)	These inguinal nodes regressed and were no longer PET-avid (1 month after treatment).	Initial staging; Treatment evaluation	(20)
18	Enlarged and FDG-avid left inguinal node (SUVmax 3.5) and right inguinal node (SUVmax 2.3)	Absence of disease on PET/CT imaging after treatment	Initial staging; Treatment evaluation	(21)

SUVmax, maximum standardized uptake value; NA, not available.

**Table 3:** Summary of SUVmax in ^18^F-FDG PET/CT of BPDCN, including our data and literature data.

Organ	Number of lesions	SUVmax
Skin (single)	5	6.7 (range 2.8–6.7)
Skin (multiple)	3	2.6 (range 1.5–2.6)
Lymph nodes	4	6.8 (range 2.3–6.8)
Bone marrow	2	9.1 (range 1.5–9.1)
Spleen	1	4.4 (range 4.4)
Breast	1	1.9 (range 1.9)
Lung	1	5.6 (range 5.6)
Sum	17	9.1 (range 1.5–9.1)

SUVmax, maximum standardized uptake value.

The ability of PET/CT to detect more lymph nodes involved in lymphoma compared with CT alone was confirmed by a previous study. The metabolic information of PET may help identify the lymph nodes that do not fulfill size criteria (25). Furthermore, 39% of patients with BPDCN had lymph node lesions (4). Our study revealed moderate-to-high FDG-avid lymph node lesions (range, 2.3–6.8); complete metabolic remission was seen after treatment in five patients. PET could distinguish benign lymph nodes from involvement by comparing the FDG uptake values before and after treatment. Furthermore, staging with PET/CT rather than CT could easily detect subcentimetric lymph nodes and assess the extent of nodal involvement (12, 13, 19).

Furthermore, 62% of patients with BPDCN experienced bone marrow infiltration (4). However, only four cases (57.1%) in our study had high uptake of FDG in bone among the seven cases with a positive marrow aspirate. Given the reported sensitivity of PET for detecting marrow involvement in non-Hodgkin lymphoma, positive bone marrow involvement is not always evident on ^18^F-FDG PET (26). This serves as a reminder that imaging alone may underestimate bone marrow infiltration, and a bone marrow biopsy should be necessary in the initial evaluation.

As observed in case number 10, the patient had plaques on the right cheek, and ^18^F-FDG PET/CT incidentally detected a hypermetabolic breast lesion with proven BPDCN involvement. This discovery changed the patient’s treatment strategy, and she remains in complete remission without relapse at 34 months since the initial diagnosis (8). Therefore, PET/CT may have remarkable advantages in detecting potential lesions in the whole body and providing guidance on biopsy location.

**Figure 3:**
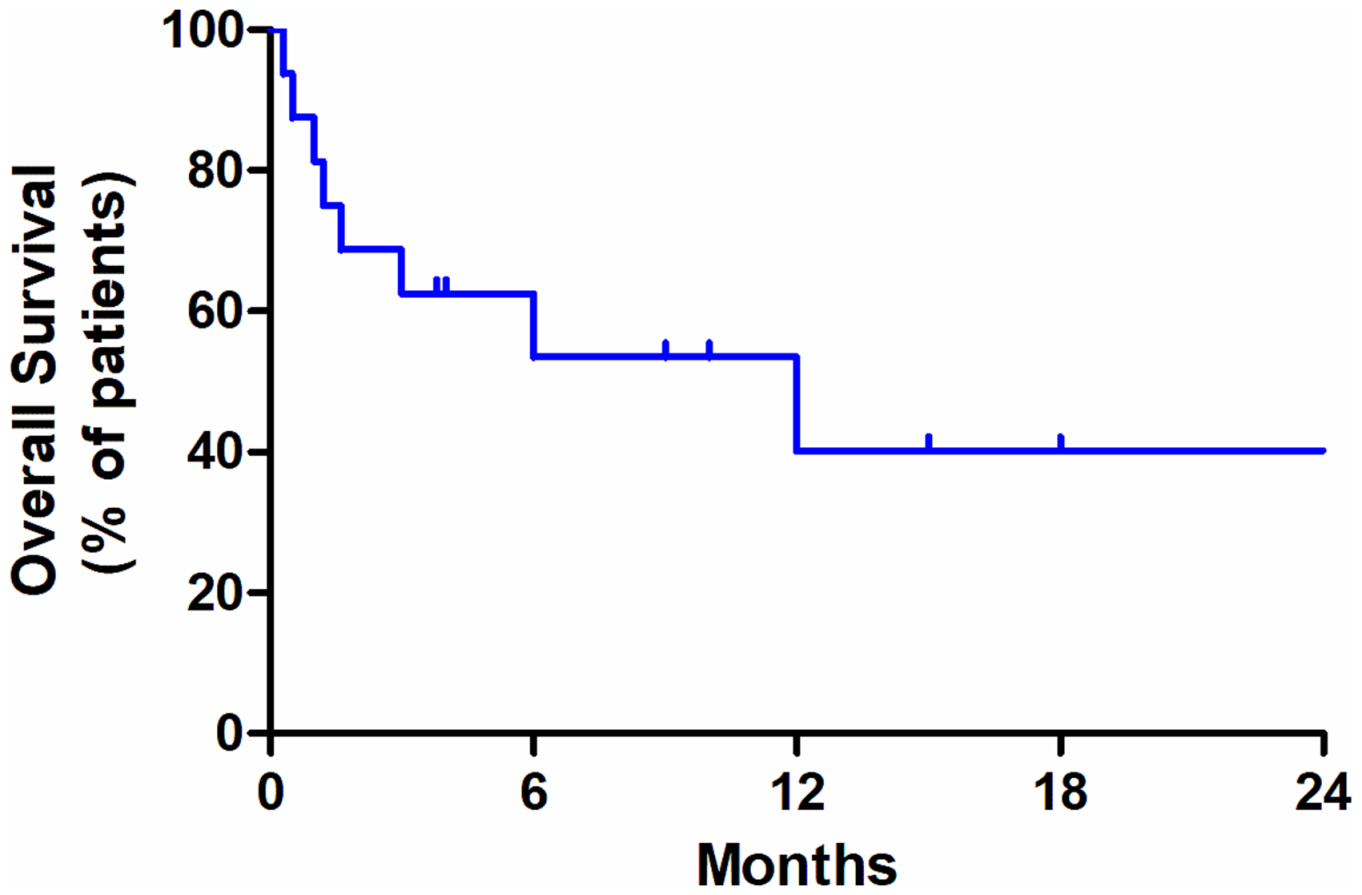
Kaplan–Meier survival curve.

The data regarding treatment were available for 15 patients. Treatment consisted of NHL-like chemotherapy in one patient, ALL-like regimens in four patients, AML-like regimens in two patients, and ALL-like treatment, followed by PBSCT in one patient, multiple chemotherapeutic regimens in two patients, radiotherapy in one patient, SL401 in one patient, and palliative treatment in three patients. A total of 7 of 18 cases reported the follow-up data of PET/CT, revealing metabolic reduction or remission after treatment. In a clinical trial regarding the activity of SL-401 in patients with BPDCN, ^18^F-FDG PET/CT was performed before treatment, after 1 month, and then every 3 months and at times of disease progression (21), demonstrating that ^18^F-FDG PET/CT might serve as a tool in the efficacy evaluation and follow-up of BPDCN. As shown in Hodgkin lymphoma, higher PET parameters at baseline were associated with a poor prognosis (27). Prognostic data were available for 16 patients in our cohort. The median OS in this cohort was 12.0 months (range: 0.5–34 months), and the median follow-up time was 10.0 months (Figure 3). Among these, 10 cases reported SUV_max_ of lesions at the same time. Interestingly, five cases out of eight patients with SUV_max_ > 2.5 died within 2 months of diagnosis, whereas two other cases with SUV_max_ < 2.5 survived within 10 and 34 months of follow-up. In our BPDCN study, we observed that patients with SUV_max_ > 2.5 might experience early death. This suggested that SUVmax might be associated with the prognosis of BPDCN. This study was novel in reporting that FDG uptake might be associated with BPDCN prognosis. However, the data were not sufficient for further statistical analysis.

Scholars suggested that CT or PET/CT should be included in the initial evaluation of a patient with suspected BPDCN (3). In our cases and reported cases, ^18^F-FDG PET/CT played a vital role in initial staging (18/18), selection of biopsy sites (5/18), and treatment evaluation (7/18). Seven cases had follow-up data using ^18^F-FDG PET/CT (2, 13, 15, 18–21), which showed that during treatment evaluation or clinical follow-up, ^18^F-FDG PET/CT could assess disease remission through the resolution of hypermetabolic lesions or absence of disease (Table 2). In our study, ^18^F-FDG uptake substantially increased in lesions in all 18 cases, indicating a sensitivity of 100%. Few studies summarized the CT findings of BPDCN (7, 18), and no study focused on ^18^F-FDG PET/CT imaging features of BPDCN. However, the number of cases included in our study was relatively small due to the low incidence rate of the disease. The institutional and literature-derived data were collected retrospectively, which may introduce potential biases and incomplete data. Irrespective of its advantages over either PET or CT alone, PET/CT is an ideal imaging technique for detecting BPDCN involvement. Additional studies should be performed to address the usefulness and cost-effectiveness of ^18^F-FDG PET/CT in the clinical practice of BPDCN.

## Conclusion

Our case series and cases from the literature demonstrated the utility of ^18^F-FDG PET/CT in the diagnosis, staging, prognosis, and treatment follow-up of BPDCN. Early recognition of this rare malignancy on imaging can expedite diagnosis and facilitate early treatment.

## Data Availability

The raw data supporting the conclusions of this article will be made available by the authors, without undue reservation.
